# Translation of bi-directional transcripts enhances MHC-I peptide diversity

**DOI:** 10.3389/fimmu.2025.1554561

**Published:** 2025-03-17

**Authors:** Filip Zavadil, Tomas Henek, Justine Habault, René Chemali, Maria Camila Tovar-Fernandez, Chrysoula Daskalogianni, Laurence Malbert-Colas, Lixiao Wang, Sivakumar Vadivel Gnanasundram, Borek Vojtesek, Lenka Hernychova, Sebastien Apcher, Robin Fahraeus

**Affiliations:** ^1^ RECAMO, Masaryk Memorial Cancer Institute, Brno, Czechia; ^2^ Inserm UMRS1131, Institut de Génétique Moléculaire, Université Paris Cité, Hôpital St. Louis, Paris, France; ^3^ UMR 1015 Immunologie des tumeurs et immunothérapie contre le cancer, B2M, Gustave Roussy, Université Paris Sud, Villejuif, France; ^4^ Department of Medical Biosciences, Umeå University, Umeå, Sweden; ^5^ Laboratory of Growth Regulators, Institute of Experimental Botany, The Czech Academy of Sciences, Olomouc, Czechia

**Keywords:** MHC-I epitope, Pioneer Translation Products, bi-directional transcripts, bi-directional translation, reverse strand antigenic peptides

## Abstract

Antisense transcripts play an important role in generating regulatory non-coding RNAs but whether these transcripts are also translated to generate functional peptides remains poorly understood. In this study, RNA sequencing and six-frame database generation were combined with mass spectrometry analysis of peptides isolated from polysomes to identify Nascent Pioneer Translation Products (Na-PTPs) originating from alternative reading frames of bi-directional transcripts. Two Na-PTP originating peptides derived from antisense strands stimulated CD8^+^ T cell proliferation when presented to peripheral blood mononuclear cells (PBMCs) from nine healthy donors. Importantly, an antigenic peptide derived from the reverse strand of two cDNA constructs was presented on MHC-I molecules and induced CD8^+^ T cell activation. The results demonstrate that three-frame translation of bi-directional transcripts generates antigenic peptide substrates for the immune system. This discovery holds significance for understanding the origin of self-discriminating peptide substrates for the major histocompatibility class I (MHC-I) pathway and for enhancing immune-based therapies against infected or transformed cells.

## Introduction

Peptides presented on major histocompatibility class I molecules (MHC-I) forms a fundamental aspect of the immune system’s capacity to detect and destroy infected or transformed cells (1). The pathways leading to the processing, presentation and detection of these peptides are targets for immune evasion by viruses and tumour cells (2, 3). Much effort is being invested in new cancer therapeutics aiming to enhance the immune system’s capacity to eliminate tumour cells presenting neoantigens, including vaccines, immune checkpoint inhibitors or CAR T cells (4). However, less effort has been devoted to the origin of neoantigens and if tumour cells have evolved mechanisms to interfere with their synthesis.

Most of the known viral and tumour antigens, including mutational neo-epitopes, originate from traditional well-characterized coding regions of the viral or human genomes. However, studies have identified viral and tumour-specific noncanonical open reading frames (ORFs), including those derived from noncoding, non-annotated, and non-AUG-initiated isoforms (5, 6). Although the functions of noncanonical ORFs remain poorly understood, emerging evidence indicates that the encoded polypeptide products play a crucial role in MHC-I antigen presentation, particularly in the context of viral infections and cancer, but also in healthy, uninfected cells (7–9). Together with recent studies showing that MHC-I antigenic peptides originate from unexpected sources, such as microproteins (10) and long non-coding RNAs (lncRNAs) (11, 12), these findings underline that noncanonical translation events contribute significantly to the generation of antigenic peptides.

The importance of a better understanding of neoantigen synthesis is highlighted by the fact that peptide substrates for the MHC-I pathway originate from non-AUG translation initiation of pre-mRNAs (13, 14). The synthesis of these Pioneer Translation Products (PTPs) is carried out by a translation event that is spatiotemporally distinct from the canonical translation giving rise to full-length proteins and blocking mRNA export from the nucleus to the cytoplasm markedly enhances neoantigen synthesis (15–17).

Bi-directional transcription is a common phenomenon and it has been estimated that approximately three quarters of human genes have corresponding antisense transcripts (18–21). A large proportion of antisense promoters are activated by immune-responsive factors during immune challenge (22). These transcripts primarily generate unstable non-coding RNA (ncRNA) controlling the stability and translation of the sense strand *in cis* or *in trans*. However, whether antisense transcripts are translated and what function they may serve remain largely unknown.

In this study we have identified endogenous nascent-derived PTPs (Na-PTPs) originating from non-exon coding sense and antisense transcripts that are presented to immune cells via the MHC-I pathway. This shows that antisense transcripts do not simply generate non-coding regulatory RNAs but are also translated to provide a source of peptide substrates for the MHC-I pathway. Uncovering the origin of Na-PTPs and what controls their synthesis will have profound implications for a better understanding of immune surveillance, for predicting neoantigen presentation and for the manipulation of neoantigen expression that together will advance the development of immune-based therapies.

## Results

### Identification of nascent peptides derived from translation of pre-mRNAs

**Figure 1:**
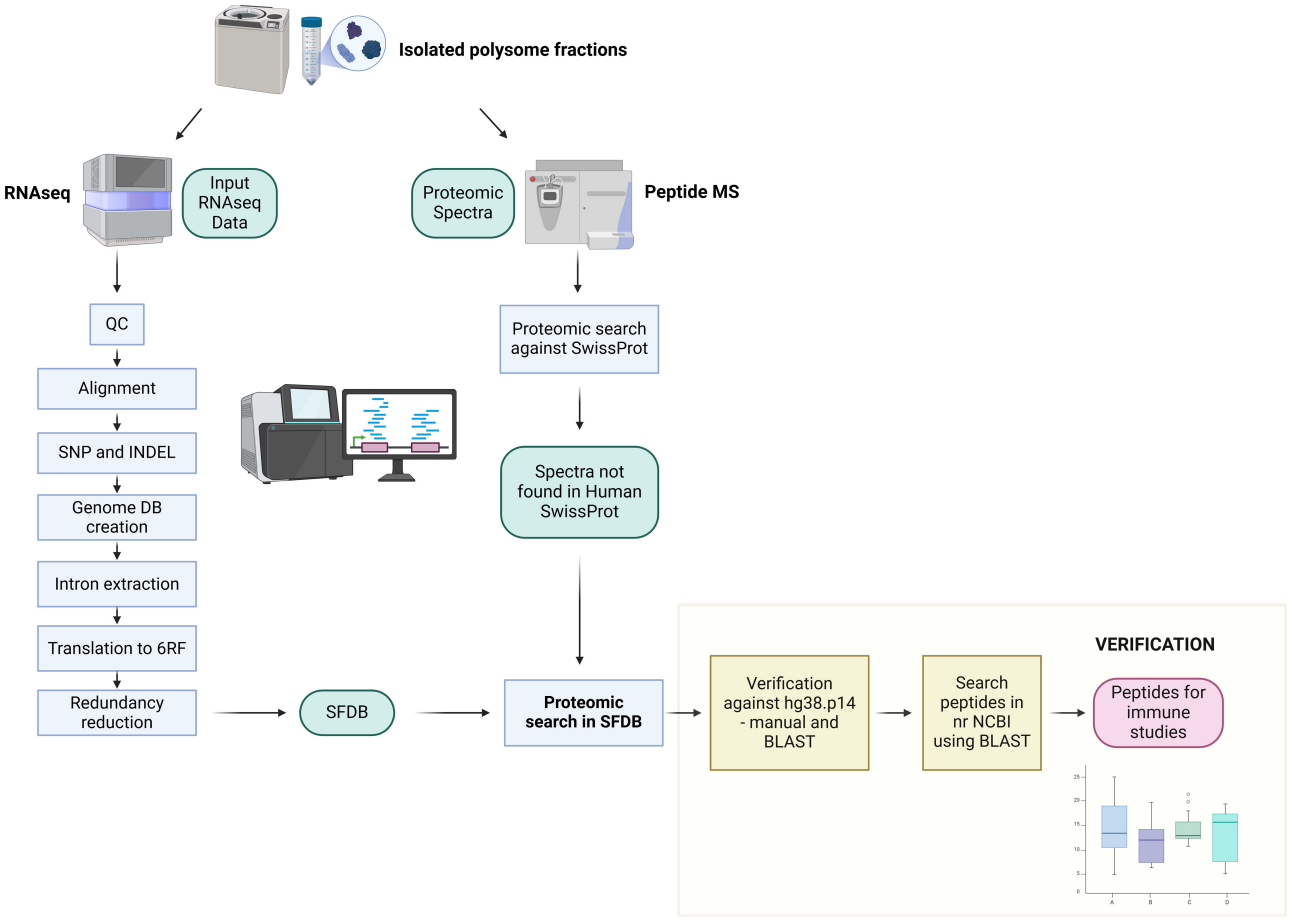
Identification of Nascent Pioneer Translation Products (Na-PTPs). The bioinformatics workflow for analyzing nascent peptides derived from non-exonic sequences is illustrated. Polysomes from cells treated with cyclohexamide to freeze the nascent peptides on ribosomes were isolated and subjected to RNA sequencing (RNA seq.) and mass spectrometry (MS) analysis. QC: quality control of input reads. Alignment: alignment of reads to reference genome hg38.p14. SNP and INDEL: detection of single nucleotide polymorphism and insertions/deletions by VarScan2. Genome creation: creation of the sample-specific genome sequence with incorporated mutations. *Intron extraction*: extraction of the intron sequences for each sample. Translation to 6 Reading Frames: translation into six possible reading frames and creation of sample specific database. Redundancy reduction: sample specific databases were merged and redundancy reduction for the same sequences was applied. SFDB: The resulting six frame proteomic database: Measured MS data were first searched against Human taxonomy. Spectra unassigned in this search were then used for the search against the SFDB. Verification: candidate sequences were verified against the hg38.p14 database for multiple occurrences in genome and blasted against Human SwissProt database to exclude previously described peptides. See also **Supplementaryay Figures S1, S2**.

In order to better understand the origin of endogenous antigenic peptide substrates for the MHC-I pathway we set out to analyze Nascent Pioneer Translation Products (Na-PTPs) derived from various RNA regions. We specifically focused on Na-PTPs arising from non-exonic sequences to discriminate from peptides that might originate from full-length proteins. For that purpose, polysome fractions were isolated from lysates of Hek293 or H1299 cells treated with cycloheximide to freeze the nascent peptides on the ribosomes (23). The polyribosomes were then subjected to RNAseq and to mass spectrometry analysis (**Figure 1**).

To determine nascent peptides derived from non-exon sequences we first carried out an RNAseq quality control of the input reads for all samples (see Material and Methods). Out of the 413 million reads obtained, 75-80% aligned with the reference genome hg38.p14 (ENSEMBL database) that is in line with what is standard for RNAseq samples. Other quality control parameters (such as GC content, adapter content, overexpressed sequence) were evaluated and found to be satisfactory. The numbers of aligned reads were distributed between 15,3 and 27,3 million reads per sample. Single nucleotide polymorphisms (SNPs) and insertions/deletions (INDELs) were detected in the aligned reads. The number of SNPs and INDELs varied between 75 and 145 thousand events with a p-value < 0.01 with a consistent proportion of SNPs and INDELs across all RNAseq samples. Identified mutations were used for modification of the reference genome hg38.p14 enabling the creation of a sample-specific genome. The gtf file containing information about introns and exons annotated in human genome hg38.p14 was used as input for a custom script designed to extract non-exon sequences from our sample specific genomes. These sequences were then translated into peptide sequences across all six reading frames, merged and redundant sequences were removed. The resulting sequences were converted into a FASTA file for the creation of a **s**ix **f**rames proteomics **d**ata**b**ase (SFDB).

**Figure 2:**
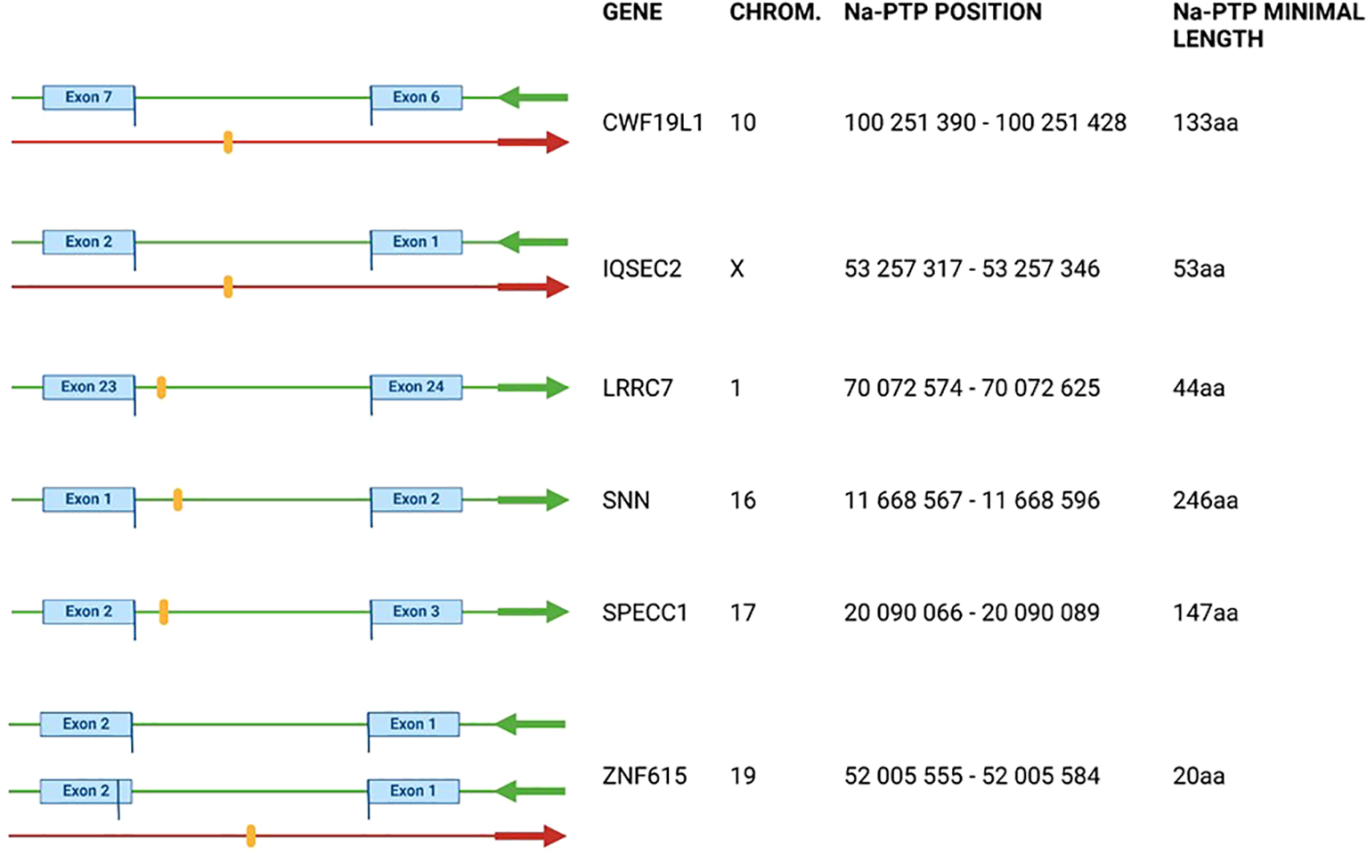
Visualization of detected Nascent Pioneer Translation Products (Na-PTPs) identified by proteomics search and manually verified by human genome reference hg38.p14 and SFDB databases. Detected peptides are shown as orange stripes. Green arrows indicate direction of open reading frames and red arrows the direction of translation of PTPs from antisense strand corresponding to genes CWF19L1, IQSEC2 and ZNF615. For ZNF615 two splice variants with different origin of exon are shown. The exact position of the peptide in the chromosome (column “Chrom”) is shown in the “Na-PTP position” column. Peptide TLLTETGAGR from the antisense strand of ZNF615 was also detected in H1299 cells. See also **Table 1** and **Supplementary Figures S1, S2**.

Peptides isolated from polysomes were analyzed by tandem mass spectrometry using an Orbitrap Fusion and evaluated using Peaks Studio 10 software (**Supplementary Figures S1, S2** and Material and Methods). The measured spectra were searched against the SwissProt database to exclude already annotated peptides. Those not corresponding to the SwissProt database were matched against the SFDB. Thirty-two nascent peptides were classified as non-exon derived Na-PTP candidates from the sense, or the antisense strand. The identified peptides were verified manually and by BLAST searches against the hg38.p14 reference genome from ENSEMBL database and blasted against nr NCBI database (**Figure 1**). Twenty-six peptides were excluded due to possible multiple occurrences in the reference genome, or annotations in the human proteome. The remaining six candidates had only a single unique non-exon occurrence and were annotated relative to the associated gene (**Figure 2**, **Supplementary Figure S3**).

### Description of verified Na-PTP peptides from non-coding sequences

The annotation information of the six non-exon derived Na-PTPs is summarized in **Figure 2** with position of the peptide-encoding sequences in the reference genome (chromosome, chromosome coordinates), the direction of translation compared to translation of the associated main open reading frame (ORF) and the predicted minimal length of the Na-PTPs. Further information about the genomic sequence of the detected peptides is provided in **Supplementary Figure S3**. The position of the non-exon derived peptides was graphically presented in orange together with nearby exons and introns with their respective start/end positions on the given chromosome. Three peptides were found to originate from the opposite strand as compared to the main ORF of genes CWF19L1; IQSEC2; ZNF615, respectively. The site of translation initiation cannot be predicted but it has been shown that translation termination of PTPs follows the same stop codon usage as for full length proteins and based on this we estimated the minimal length of the Na-PTPs to between 20 and 245 amino acids. It should be noted that peptides derived from introns, from alternative reading frames as well as from the reverse strand are all annotated as non-exon derived Na-PTP peptides.

We conducted a similar analysis of Na-PTPs in human carcinoma H1299 cells. This cell type differs from the human embryonic kidney HEK293 cells. However, surprisingly, despite using different mass spectrometers (Orbitrap Fusion and timsTOF SCP), we identified one Na-PTP (TLLTETGAGR) that was also detected in HEK293 cells and originates from the antisense strand of ZNF615 (**Supplementary Figure S2**).

### Verification of translated antisense transcripts

**Figure 3:**
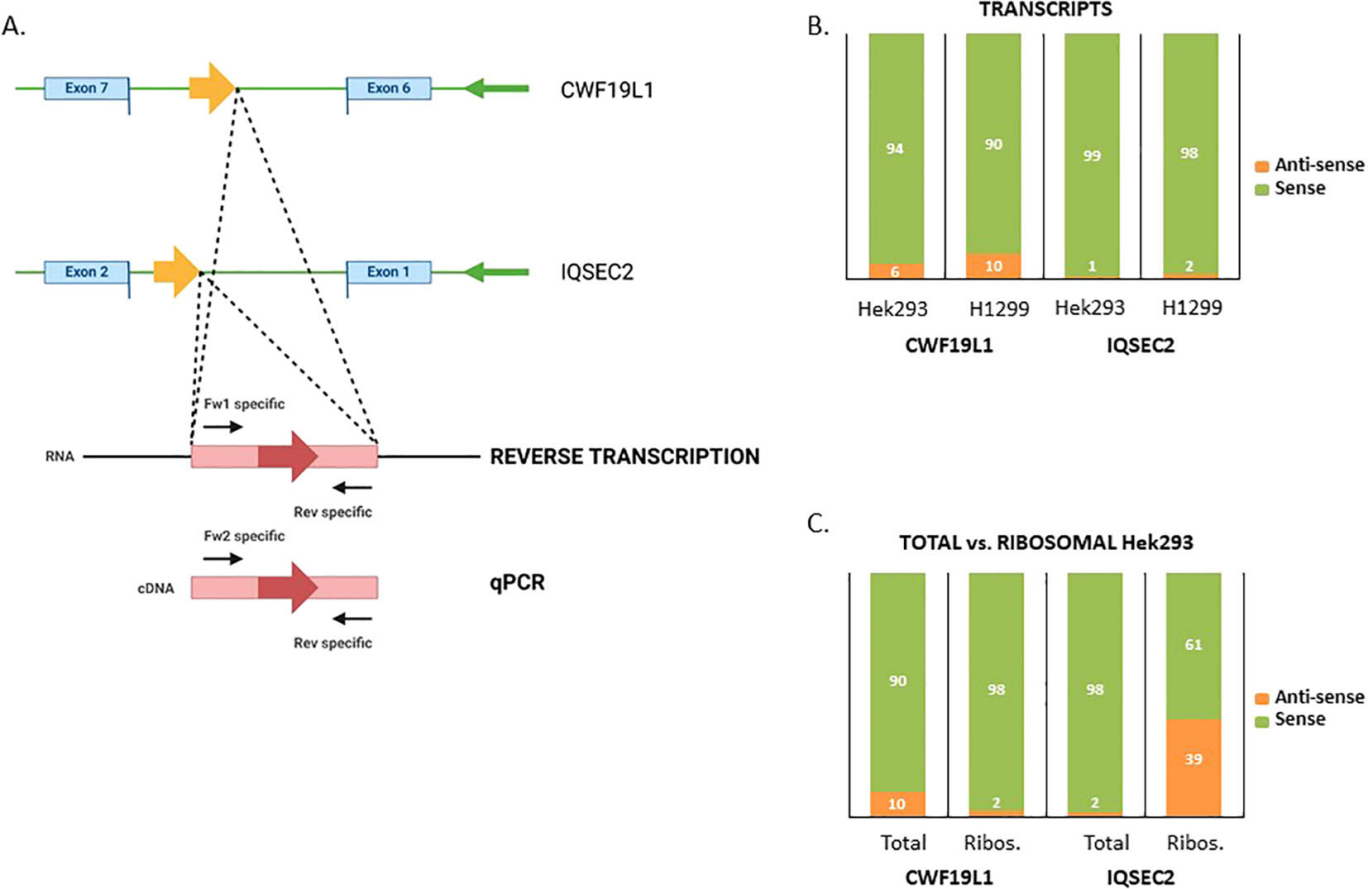
Verification of translated antisense transcripts. **(A)** Forward and reverse primers were used to estimate the expression of the sense and antisense strands of the IQSEC2 and CWF19L1 genes in Hek293 and H1299 cell lysates. **(B)** The relative amount of sense and antisense CWF19L1 and IQSEC2 transcripts. **(C)** Comparing total *vs*. ribosome-associated levels of sense and antisense transcripts for CWF19L1 and IQSEC2.

To confirm that the detected Na-PTPs originate from the opposite RNA strand, we verified the presence of antisense transcripts corresponding to the IQSEC2 and CWF19L1 genes in Hek293 cells, as well as in H1299 cells, using qPCR analysis (**Figure 3A**). Due to different sets of reverse and forward PCR primers (**Supplementary Table S1**) the relative amount of sense *vs*. antisense transcripts can only be estimated. Nevertheless, in line with what has previously been shown, our data suggest that approximately 94% of the transcripts of CWF19L1 were from the sense strand and 6% from the antisense strand in Hek293 cells and the corresponding numbers in H1299 cells were 90% vs. 10%. For the IQSEC2, less than 1% were estimated as antisense transcripts in Hek293 cells and less than 2% in H1299 cells (**Figure 3B**). Next, we aimed to estimate the proportion of ribosome-associated RNA relative to the total transcript levels. When we compared the total amount of transcripts *vs*. the proportion of ribosome-associated RNA we observed differences. For CWF19L1 the relative amount of antisense transcripts associated with ribosomes, was less than 2% in Hek293 cells. For IQSEC2 the relative amount of antisense RNAs linked to ribosomes was 39% (**Figure 3C**). This difference in the relative amount of antisense RNAs associated with ribosomes can be explained by differences in the relative number of spliced mRNAs being translated.

### Peptides translated from the reverse strand activate CD8+ T cells from healthy donors

**Figure 4:**
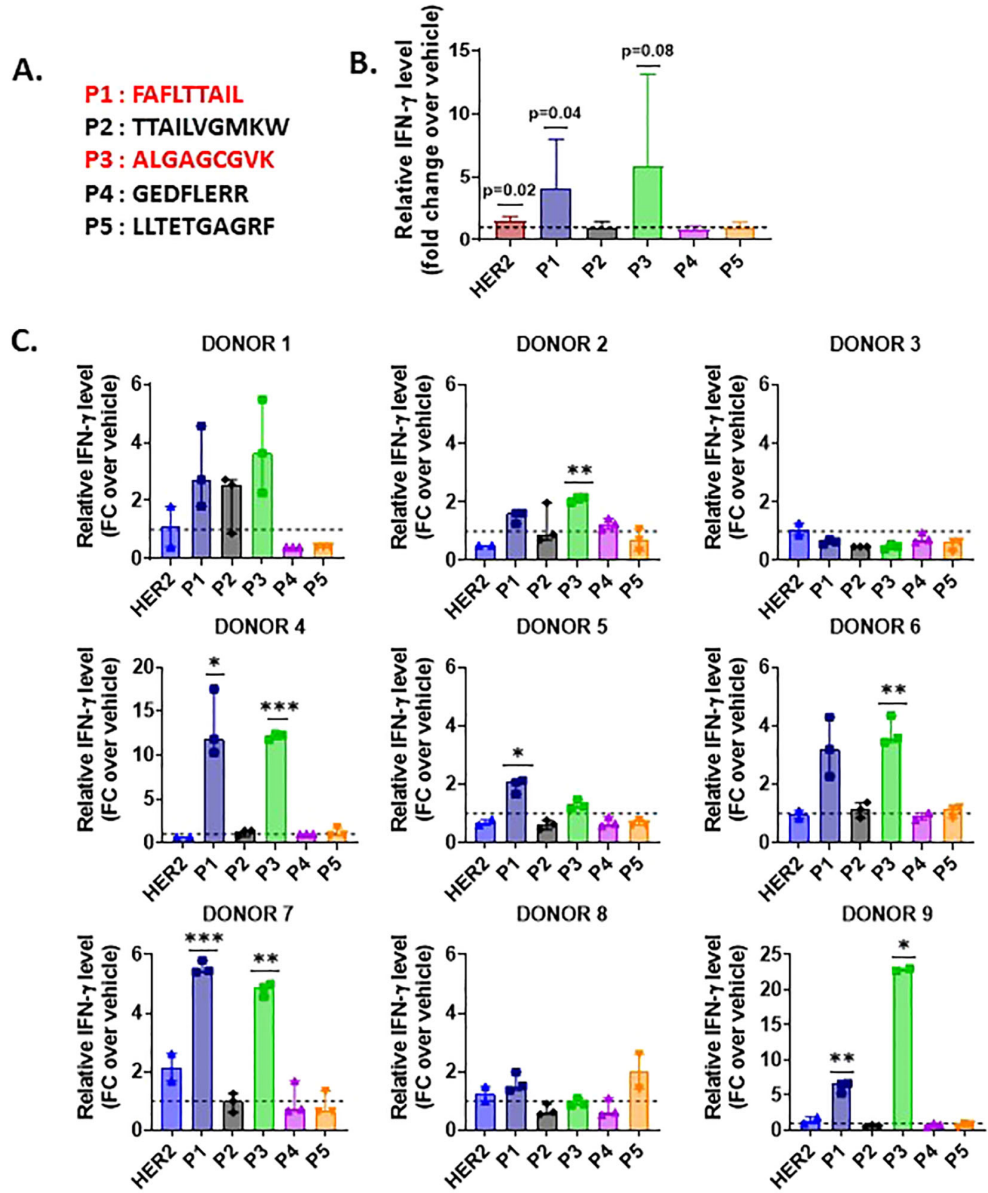
IFN-γ production by CD8^+^ T cells stimulated with Na-PTP peptides in PBMCs from nine healthy donors. **(A)** Sequences of the five peptides (P1 to P5). **(B)** Mean fold change (FC) in relative IFN-γ levels for each peptide (P1 to P5) and the positive control (HER2) across all donors. **(C)** Individual bar charts displaying the relative IFN-γ levels (fold change (FC)) for each donor across peptide conditions, compared to the baseline (dashed line at FC = 1). Statistical significance was assessed using a one-sample t-test, comparing the observed mean IFN-γ level for each peptide to a theoretical mean of 1. P values < 0.05 are indicated on the figure.

As peptides derived from non-coding sequences are presented to the MHC-I pathway, we next sought to investigate if Na-PTPs can induce specific immune responses. For that purpose, we tested the potential of antigenic epitopes within 8- to 14-mer peptides across ribosome-associated Na-PTPs using the NetMHCpan software for their affinity to MHC-I molecules (24). Several Na-PTP peptides indicated binding affinity, suggesting potential presentation by at least one representative allele from the HLA-A or HLA-B supertypes. Out of five selected candidate peptides (**Table 1**), one represented sense and four represented antisense-derived peptides. These were then tested for their immunogenicity using peripheral blood mononuclear cells (PBMCs) derived from nine healthy donors. PBMCs were individually stimulated twice with each peptide and the activation of peptide-specific CD8^+^ T cells was evaluated by measuring IFN-γ production through an ELISA assay. The HER2 peptide served as a positive control as it triggers an IFN-γ response in immune assays (25) while sterile-filtered water was used as a negative control to establish baseline cytokine production. Remarkably, the two peptides P1 (FAFLTTAIL) and P3 (ALGAGCGVK) that originate from antisense transcripts elicited the most robust CD8^+^ T cell response (**Figures 4A–C**, **Supplementary Figures S4**–**S6**). Two antigenic peptides (P1: FAFLTTAIL and P2: TTAILVGMKW) from the Na-PTP derived from the reverse strand of the CWF19L1 gene were predicted to be immunogenic but only the P1 induced CD8^+^ T cell proliferation (**Table 1**). In summary, the observed immune responses against P1 and P3 across different donors highlight the potential of these peptides to activate CD8^+^ T cells. This finding shows that antisense-derived peptides can trigger strong immune responses, which could make them important for developing new strategies to modulate the human immune system and design therapeutic treatments.

**Table 1:** Nascent pioneer translation products (Na-PTPs) and their corresponding predicted antigenic peptides with their different lengths.

Gene	Amino acid sequence of of the different Na-PTPs	Length [aa]	Amino acid sequence of peptide predicted by NetMHC pan	Length [aa]
CWF19L1	F.AFLTTAILVGMK.W	14	FAFLTTAIL (**P1**) and TTAILVGMKW (**P2**)	9 10
IQSEC2	R.FALGAGCGVK.A	12	ALGAGCGVK (**P3**)	9
SPECC1	R.LGEDFLER.R	10	GEDFLERR (**P4**)	8
ZNF615	K.TLLTETGAGR.F	12	LLTETGAGRF (**P5**)	10

Black letters indicate amino acids identified by mass spectrometry and in red flanking amino acids. Peptide numbers (in brackets) relate to **Figure 4**. Please see also **Supplementary Figure S2** reading origin and choice of peptides.

### Peptides derived from antisense strands are presented on MHC-I molecules

**Figure 5:**
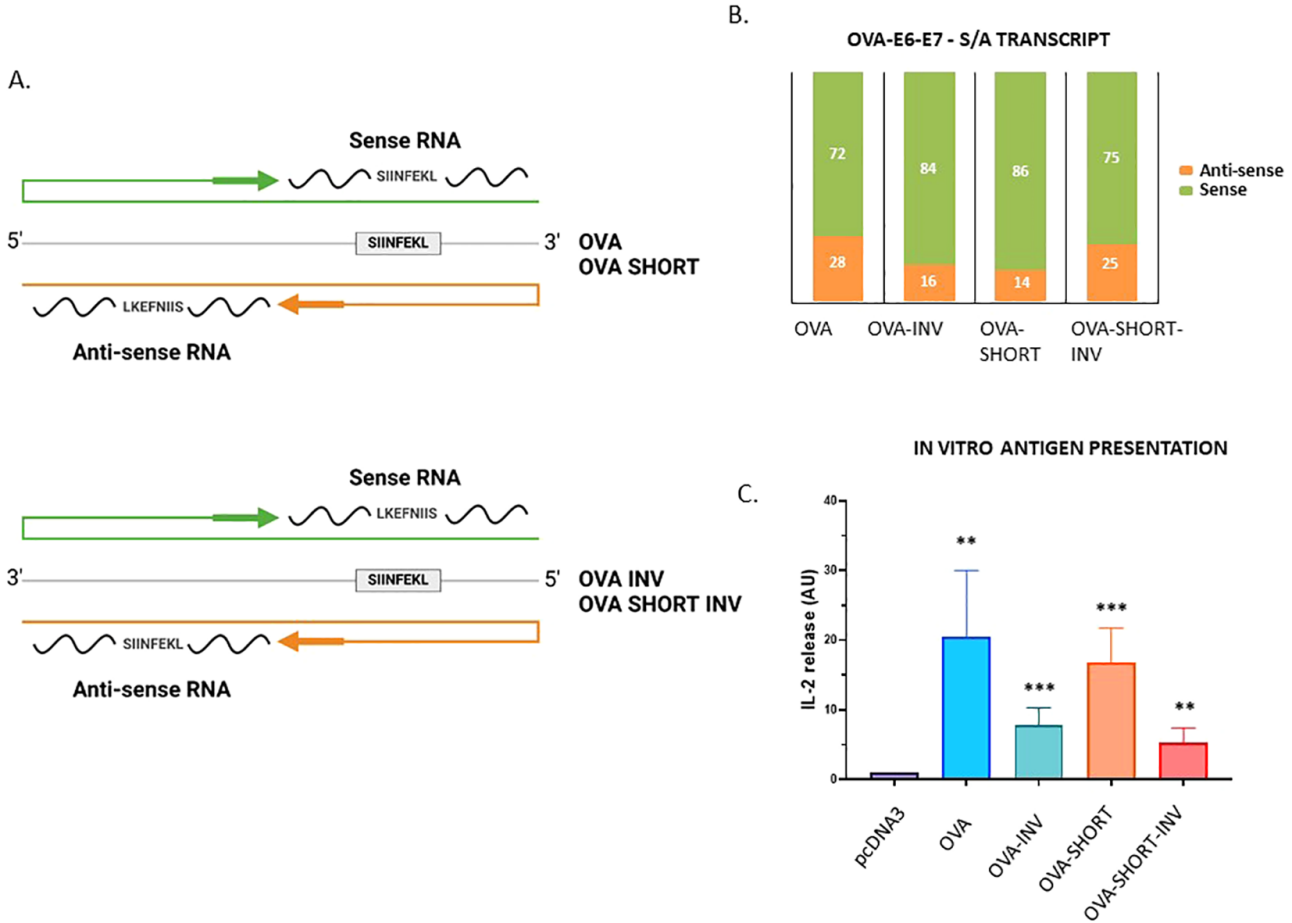
Peptide substrates derived from the antisense strand are processed and presented on MHC-I molecules. **(A)** The SL8 (SIINFEKL) peptide is derived from chicken ovalbumin (OVA) and presented on murine Kb MHC-I molecules. Four OVA constructs were generated in which SL8 was expressed from the sense or antisense strand. OVA corresponds to the full-length OVA whereas OVA SHORT corresponds to exons 5, 6 and 7. **(B)** The relative expression of the transcripts shows that the sense and antisense strands are expressed. **(C)** CD8+ T cell assay using OT-1 cells that specifically detect SL8 presented on Kb molecules shows that translation of both strands generates SL8 peptides. The data show an average of five independent experiments (unpaired t test, ***p-value <0.001, **p-value<0.005).

The presented data show that endogenous antisense transcripts are translated and that some of the encoded peptides can trigger a CD8^+^ T cell response in healthy donors. To test if peptides derived from antisense transcripts are indeed presented to the MHC-I pathway and induce CD8^+^ T cell response we generated two constructs in which the MHC-I reporter peptide SL8 (SIINFEKL) was expressed from the sense and antisense directions. The SL8 is derived from chicken ovalbumin (OVA) and presented on murine Kb MHC-I molecules and is specifically detected by OT-1 CD8+ T cells isolated from transgenic mice (26). We inserted the full-length OVA, as well as a shorter version corresponding to the three last exons 5, 6 and 7 of the OVA gene (OVA SHORT) that also encodes the SL8 peptide (position aa. 257-264) in pcDNA3.1 vectors. We also reversed these constructs so that the SL8 would be encoded from the antisense strand (OVA INV. & OVA SHORT INV, respectively) (**Figure 5A**). From the RT-qPCR data we estimated that the relative expression of the sense *vs*. antisense was approximately 72% to 28% for the OVA construct and 86% to 14% for the OVA SHORT construct. The corresponding numbers for the reverse constructs were 84% to 16% for the OVA INV. and 75% to 25% for the OVA SHORT INV. (**Figure 5B**). The OVA constructs were expressed in Hek293 cells together with the MHC-I Kb molecule. 10^6^ OT-1 cells were added 48 hours after transfection and the expression of SL8 on the Kb molecules was indirectly determined by measuring IL-2 levels in the tissue medium after 12 hours incubation. (**Figure 5C**, **Supplementary Figure S5B**). Interestingly, the SL8 peptide was expressed from the antisense strand of both reverse constructs at levels comparable to transcript levels, indicating that the synthesis of Na-PTPs is as efficient from either strand. These data show that translation of bi-directional transcripts generates antigenic peptides that are presented to the MHC-I pathway and activates a CD8^+^ T cell response.

## Discussion

Previous works using a reporter MHC-I peptide have shown that a spatiotemporally distinct mRNA translation event synthesizes non-exon derived Pioneer Translation Products (PTPs) for the MHC class I pathway (16, 23, 27). This has opened for the possibility to selectively manipulate the production of neoantigens synthesis while leaving the expression of full-length proteins unaffected. But to reach this stage, more information is needed regarding the mechanism governing PTP synthesis. Currently, our understanding of how PTPs are synthesized is limited, though it is known that translation can be initiated from non-AUG codons on pre-mRNAs (13, 23). To get a better idea of an endogenous global PTP synthesis, we analyzed endogenous nascent peptides derived from non-exon encoding sequences by isolating polysome fractions in which the nascent peptides were frozen on the ribosome. A six-frame database corresponding to exon and non-exon sequences was created and was used to match ribosome-associated peptides identified by mass spectrometry with RNASeq data of isolated polysomes. Six peptides of eight or more amino acids were selected that did not match the SwissProt data base and for which the genomic origin could be assigned with high confidence.

Together with a high stringency process to ensure that the selected peptides were indeed derived from non-exon sequences, we did not expect many hits of the same peptide. Out of the six identified peptides, three were derived from the antisense strand. Two Na-PTP peptides were shown to induce a CD8^+^ T cell response when presented to PBMCs from healthy donors. We confirmed that the synthesis of neoantigens from bi-directional transcripts are indeed presented to the MHC-I pathway by expressing the SL8 MHC-I epitope from the antisense strand of two different constructs. This finding is significant as it demonstrates that antisense strands are translated to generate MHC-I peptide substrates, highlighting a cell biological event with interesting evolutionary implications of which we know very little. It is a fascinating thought that a defined mRNA translation event, distinct from those producing full-length proteins and, presumably, carried out by a specific ribosome, forms a cornerstone in the immune system’s capacity to distinguish self from non-self. This translation event may have evolved in parallel to the canonical translation or, more likely, represents a more primitive translation that is less dependent on strict AUG codon initiation and that has been conserved and adapted to the MHC-I processing pathway to provide peptide products for immune surveillance. A guess would be that a designated translation event for producing peptides from both strands would enhance the immune system’s capacity to detect pathogen infection. In line with this reasoning, a non-canonical random translation initiation would make it more difficult for viruses to evolve messages that circumvent the synthesis of neoantigens. However, in this study, we have focused on endogenous Na-PTP substrates, and it is not known if this translation event also acts on viral RNAs. However, it is argued that introduction of cDNA constructs is sensed by the cells as virus-like infection and if so, the expression of the SL8 peptide from antisense transcripts would argue that translation of bi-directional transcripts also takes place on non-endogenous genomes. Another intriguing aspect is the observation that antisense transcripts appear to be translated in general. These transcripts are generally thought to provide non-coding RNAs (28, 29) rather than serving as a source for the synthesis of a specific class of peptides. Little is known on what controls transcription of the reverse strand except that it is more susceptible to immune stimulatory pathways (22). But if antisense transcription is directly stimulated by viral and pathogen infections remains unknown.

A better characterization of the ribosome carrying out translation of pre-mRNAs and antisense transcripts and what governs the initiation of PTP synthesis could open up for new therapeutic intervention strategies that selectively target neoantigen production. For example, a difficulty with neoantigen-based immune therapies is that we do not know if all tumour cells express the same neoantigens, or if a cancer cell expresses the same neoantigens over time (30). The possibility to specifically control PTP synthesis, either selectively, or globally, would help make existing cancer immune therapies more effective and reduce the risks of treatment resistance. Supporting this concept, recent studies have demonstrated that the antigen repertoire presented on MHC-I molecules at the surface of cancer cells can be altered by treatment with specific splicing inhibitors, positioning the spliceosome as a promising druggable target for inducing Na-PTP synthesis and enhancing anti-cancer immunity (31, 32). Furthermore, the fact that PTPs originate from six frames and from non-coding sequences helps to better assess the original coding sequence for MHC-I peptides will also help improve algorithms that predict the correlation between genetic changes in cancer cells and the presentation of neoantigens (33–35). A yet unexplored possibility is that these peptide substrates also play a role in autoimmune diseases, adding further to the potential benefits of controlling PTP synthesis.

These observations further underline that non-coding sequences constitute a source for the immune system and that neoantigens for the MHC-I pathway are derived from a far larger genetic source than was previously thought. It should be kept in mind that the six-frame database used here only correlates to the 2% of the genome that encodes for genes, and we do not know if PTPs can also be derived from intergenic sequences. Nevertheless, this potential exponential increase in peptide substrates available for MHC-I presentation challenges the classic view of central tolerance. Although PTPs induce immune tolerance (23), it remains unclear how this tolerance is generated, or whether PTPs are also produced in thymic cells. There is, of course, the possibility that the amount of PTPs is not “endless” and interestingly, we identified one Na-PTP expressed in both H1299 and Hek293 cells. Whether this expression in both cell lines is coincidental, or if a limited set of PTPs is expressed across different cell types is an important question that remains to be addressed. Additionally, it is also likely that the initial processing of PTP substrates drastically reduces the number of peptides that reaches the MHC-I pathway. Nevertheless, the potential amount of MHC-I peptide substrates available for a limited number of MHC-I molecules is staggering.

## Materials and methods

### RNA extraction and mRNA sequencing

Total RNA was precipitated with Ethanol 99.8% and purified with RNeasy Mini Kit (Qiagen). RNA samples were sent to a sequencing company (Novogene, UK). RNA quantity and quality were assessed by nanodrop and agarose gel electrophoresis. Then, the RNA library was formed by polyA capture and reverse transcription of cDNA. Next, samples were sequenced with a 150 bp paired-end sequencing strategy using Illumina PE150 technology and finally bioinformatic analysis was performed.

### Plasmids

OVA constructs were generated using the pcDNA3.1 vector as follows. The OVA and SHORT OVA plasmids, previously described (16), encode the full-length OVA cDNA lacking the first 50 amino acids and a short OVA cDNA containing exons 5–7, respectively.

To generate the inverted plasmid OVA INV, a second ApaI restriction site was introduced upstream of the OVA sequence via site-directed mutagenesis. The OVA cDNA was then excised with ApaI and ligated into the pcDNA3.1 vector in the opposite orientation. For the inverted plasmid SHORT OVA INV, a second XhoI restriction site was introduced downstream of the SHORT OVA sequence by site-directed mutagenesis. The SHORT OVA cDNA was excised using XhoI and subsequently inserted into the pcDNA3.1 vector in the reverse orientation.

Plasmid was generated using standard procedures. Restriction enzymes, T4 DNA ligase, and calf intestinal alkaline phosphatase were obtained from New England Biolabs. Purified synthetic oligonucleotides were obtained from ThermoFisher and Eurofins Genomics. Routine plasmid maintenance was carried out in the DH5α bacteria strain.

### RT-qPCR analysis

Total or ribosome-associated RNA was purified from Hek293 and H1299 cell lines with RNeasy Mini Kit (Qiagen). To evaluate the sense-antisense proportion of the CWF19L, IQSEC2 or OVA genes, reverse transcriptions were processed using specific reverse (sense) or forward (antisense) primers respectively, then quantitative PCR were performed. Sequences of the specific primers used are indicated in **Supplementary Table S1**.

### Cell culture and transfection

HEK293 (Human embryonic kidney) and H1299 (human carcinoma) cells were cultured in DMEM supplemented with 10% fetal bovine serum (FBS), 2 mM L-glutamine, and 1% Penicillin- Streptomycin. For polysome fractionation, HEK 293 cells were cultured in a 10 cm dish (1x10^6^) cells/well) at 37°C with 5% CO2. The day after seeding, transfections were performed using 15 μl of Gene Juice reagent according to the manufacturer’s protocol (Merck Bioscience). Cells were transfected with 2 μg of DNA.

### *In vitro* antigen presentation

Naive OVA257-264-specific CD8+ T-cells were isolated from the peripheral and mesenteric lymph nodes of OT-I-mice using the CD8+-isolation kit (Miltenyi Biotec). Subsequently, 1x10^6^ CD8+ T-cells were co-cultured with 10^5^ Hek293 cells previously transfected with the indicated constructs and a mouse Kb expression vector (a kind gift from C. Watts, University of Dundee, Dundee, UK). 12h later, supernatants were collected from the co- cultures and IL-2 levels were measured by ELISA using ELISA MAX™ Standard kit (Biolegend) following the manufacturer´s protocol. Signals were measured using a FLUOstar Optima (BMG Labtech) and data were analyzed using the software Optima Control v2.20R2.

### Polysome fractionation

Five–fifty percent wt/vol linear sucrose gradients were freshly cast on SW41 ultracentrifuge tubes (Beckmann) using the Gradient master (BioComp instruments) following the manufacturer’s instructions. Twenty-two hours post-treatment, cells (80% confluency) were treated with cycloheximide 100 μg/ml for 5 min at 37°C and washed twice with 1× PBS (Dulbecco modified PBS, GIBCO) containing cycloheximide 100 μg/ml. Cells were resuspended, lysed with polysome lysis buffer (100mM KCL, 50 mM HEPES KOH, 5mM MgCl2, 0.1% NP-40, 1 mM DTT, cycloheximide 100 μg/ml, pH 7.4) and spin at 2348xg for 10 min at 4°C. Lysates were then loaded on a sucrose gradient and centrifuged at 222228×g for 2 h at 4°C in an SW41 rotor. Samples were fractionated using a Foxy R1 fraction collector (Teledyne ISCO) at 0.5 min intervals (36).

### Protein extraction

Protein purifications from fractions were performed using Chloroform-Methanol precipitation. Samples were suspended with methanol 99.8% (VWR), chloroform 99.8%, (Sigma-Aldrich), and ultrapure distilled water (Invitrogen). Then, samples were centrifuged at 14.000xg for 5 min generating a white middle disc in the suspension. The upper aqueous phase was removed, and Methanol washing was performed. Precipitated proteins were suspended in LDS sample buffer 4x (Life Technologies) and sample reducing agent 10x (Life Technologies), heated for 10 min at 95°C, and loaded in Sodium dodecyl sulfate-polyacrylamide gel electrophoresis (SDS-PAGE) at 10%. The samples were subjected to electrophoresis using MOPS SDS running buffer 20x (Invitrogen). Gels were then stained with Coomassie brilliant blue R-250 staining solution (BioRad) for 2 h and afterward, washed out with 40% (v/v) ethanol (VWR), 10% (v/v) glacial acetic acid (Sigma-Aldrich), and 50% (v/v) Ultrapure distilled water (Invitrogen).

### In-gel digestion

Bands were excised out of the gel, washed with deionized water, cut into small pieces, and decolored with a freshly prepared 200 mM solution of ammonium hydrogen carbonate (NH4HCO3, pH 7.8) in 40% (v/v) acetonitrile for 20 min at 30°C and equilibrated in 50 mM NH_4_HCO_3_ (pH 7.8) in 5% (v/v) acetonitrile for 30 min at 30°C. The supernatant was removed and the gels were dehydrated with acetonitrile. The supernatant was removed and the samples were reduced by the addition of 10 mM DTT for 60 min at 60°C, followed by alkylation with 55 mM iodoacetamide in the dark for 45 min at room temperature. The supernatant was removed and the gel pieces were washed three times with equilibration buffer and dehydrated with acetonitrile. Trypsin digestion was carried out at 37°C overnight using sequencing-grade trypsin (Promega). Digested peptides were extracted using acetonitrile, vacuum dried, and desalted using C-18 micro spin columns (Harvard Apparatus) according to the manufacturer´s guidelines.

### LC-MS/MS analysis

Before mass spectrometry analysis, the evaporated peptide samples were dissolved in 2% acetonitrile with 0.05% aqueous TFA. LC-MS/MS analysis was carried out using an Orbitrap Fusion™ mass spectrometer (Thermo Fisher Scientific) with a New Objective digital PicoView 565 nanospray source (Scientific Instrument Services) coupled to a DionexTM UltiMateTM 3000 RSLC Nanoliquid chromatograph. The peptides were loaded into an Acclaim PepMapTM 100 nano trap column (nanoViperTM C18, 0.3 × 5 mm, 5 µm particle size, 100 Å pore size; Thermo Fisher Scientific); loading solvent 2% (v/v) acetonitrile with 0.05% (v/v) aqueous TFA at flow rate 5 μl/min. The trap column was directly connected to an Acclaim PepMapTM RSLC C18 analytical column (nanoViperTM 75 µm × 25 cm, 2 µm particle size, 100 Å pore size, Thermo Fisher Scientific) kept at 50°C.

In the first pilot experiment the samples were eluted using 64 min gradient which went from 2% B to 40% B linearly, followed by washing step at 98% B for 15 min. In subsequent projects the tryptic peptides were eluted with a 36 min linear gradient from 2-25% B and a 5 minutes gradient from 25-60% B, followed by a 7 min wash step with 98% B, and 27 min of equilibration with 2% B. Mobile phase A was composed of LC-MS grade water and 0.1% formic acid (FA) while B was acetonitrile 80% with 0.1% aqueous FA (v/v). The flow rate was 300 nl/min. The Orbitrap mass analyzer was operated in positive ion mode. The master scan was acquired at resolving power settings of 120,000 (FWHM @ m/z 200). In the pilot experiment the precursor mass range was set to 350-2000 m/z and in the subsequent projects the precursor mass range was 350-1400 m/z. The peptide precursors, selected based on their intensity in the MS scan, were fragmented using collision-induced dissociation (CID) with the normalized collision energy setting at 35%. The peptide fragments generated via CID were detected in an ion trap (rapid scan rate). MS and MS/MS data were recorded in profile and centroid data types, respectively.

LC-MS/MS analysis of the H1299 cells was carried out using a timsTOF SCP (Bruker Daltonics) mass spectrometer with captiveSpray (Bruker Daltonics) source and Nanoelute 2 (Bruker Daltonics) liquid chromatograph. The peptides were loaded into an Acclaim PepMapTM 100 nano trap column (nanoViperTM C18, 0.3 × 5 mm, 5 µm particle size, 100 Å pore size; Thermo Fisher Scientific); loading solvent 0.1% (v/v) aqueous FA. The trap column was directly connected to an Aurora Ultimate (25cm x 75µm) separation column kept at 50°C. The samples were eluted with a 64 min linear gradient from 2-20% B and a 6 min gradient from 20-48% B, followed by a 5 min wash step with 98% B. The columns were automatically equilibrated before the measurement. Mobile phase A was composed of LC-MS grade water and 0.1% formic acid (FA) while B was 100% acetonitrile with 0.1% FA (v/v). The timsTOF mass spectrometer was operating in PASEF (Parallel Accumulation and Serial Fragmentation) mode with 100ms ramp time for 10 PASEF ramps per window. Scan were acquired for 100-1700 m/z with precursors for fragmentation being further filtered using a polygonal filter in mobility to mass over charge plane effectively excluding singly charged ions. Selected precursors were fragmented using CID.

The mass spectrometry data have been deposited to the Proteome Xchange (PX) Consortium (37) via Proteomics Identifications (PRIDE) (38) partner repository with the dataset identifier PXD057573; Username: reviewer_pxd057573@ebi.ac.uk; Password: RrEKgua4Zc3B.

### Bioinformatics analysis of RNAseq, and creation peptide database

Sequencing reads from RNAseq were evaluated for quality control by FastQC [http://www.bioinformatics.babraham.ac.uk/projects/fastqc/] (**Figure 1**) and aligned to reference genome hg38.p14 (39) by TopHat2 (40). BAM files which were obtained were sorted (by samtools (41)) and used for detect variants in DNA by VarScan2 (42).

Detected variants were incorporated into genome sequence by TransPem [https://www.mou.cz/document/file/5360?dl=0] software for creation of the sample specific genome. Custom made script was used for extraction of all possible intron sequences via gtf file from ENSEMBL database hg38.p14. Introns were translated into corresponds peptide sequence database in six possible reading frames (f0, f1, and f2 for forward strain; r0, r1, and r2 for reverse strain) by custom script. The sequences from all samples were merged with respect to the frame and filtered based on redundancy. The resulting proteomic database (SFDB = six frame proteomics database) was used for proteomic analysis.

### Proteomic data processing

Proteomic data analysis was performed with Peaks Studio 10.6 (Bioinformatics Solution Inc.). Raw files from all samples (**Supplementary Table S1**) were analyzed in one search. The search was performed with 10 ppm precursor mass error tolerance and 0.6 Da fragment mass error tolerance. Search was for trypsin specific peptides with up to 3 missed cleavages. Carbamidomethylation (+57.021 Da) was considered a static modification on cysteine. Oxidation (+15.995 Da) on methionine and acetylation (+42.011 Da) on protein N-term were considered as variable modifications. Databases used for the search were SwissProt (TaxID=9606, validated fasta, updated 04/19/2021, # sequences 42,153; taxonomy: Homo sapiens), translated sequence from Glob-SL8-PTC-His construct, and cRap protein (ftp://ftp.thegpm.org/fasta/cRAP) databases. Final result was filtered to match 1% peptide FDR. Unassigned spectra from this search were then used for a search against the intron peptide sequence databases generated from intron nucleotide sequence. This search was for semi-tryptic peptides with up to 3 missed cleavages. Carbamidomethylation (+57.021 Da) on cysteine, oxidation (+15.995 Da) on methionine, and acetylation (+42.011 Da) on protein N-term were considered as variable modifications. Error tolerance was the same as initial search. SFDB (previous section) was used for proteomic search. Each reading frame was searched separately.

### Verification of intron peptides

Potential intron peptides which were identified were verified in current version of Ensembl database hg38.p14 for duplication/multiple location in genome or possible conflicts with older version of Ensemble database and against non-redundant protein sequences (nr NCBI) database whether they are not already annotated. Data acquired from H1299 cells were analyzed using Peaks 11(Bioinformatics Solution Inc.). The search was performed with 10 ppm precursor mass error tolerance and 0.05 Da fragment mass error tolerance. Search was for Semi-tryptic peptides with up to 3 missed cleavages was considered a static modification on cysteine. Oxidation (+15.995 Da) on methionine, acetylation (+42.011 Da) on protein N-term, Carbamidomethylation (+57.021 Da) on cysteine and Phosphorylation (+79.97 Da) on either serine, threonine or tyrosine were considered as variable modifications. Databases used for the search were SwissProt (TaxID=9606, validated fasta, updated 04/19/2021, # sequences 42,153; taxonomy: Homo sapiens) set as contaminant database and previously found intron peptides in HEK293. Final result was filtered to match 1% peptide FDR.

### PBMC isolation, culturing, and peptide stimulation for immune response assays

Human peripheral blood mononuclear cells (PBMCs) were isolated from buffy coats obtained from 9 donors (EFS Saint-Louis) using a Ficoll gradient method. Briefly, 5 ml of buffy coat was diluted with 35 mL of PBS (Gibco™) and layered onto 15 ml of Lymphosep (Biowest). The samples were centrifuged at 600 × g for 30 minutes without brake. After centrifugation, the PBMC layer was carefully collected, resuspended in RPMI (Gibco™), and centrifuged at 200 × g for 10 min to remove platelets. The PBMCs were then washed with 30 ml of RPMI and centrifuged at 500 × g for 5 min. Finally, the PBMCs were resuspended in 10 ml of complete medium: RPMI 1640 supplemented with 10% SVF, 2 mM glutamine, 50 U/ml Penicillin-Streptomycin, 10 mM HEPES, and 0.05 mM β-mercaptoethanol. Cell count and viability were assessed using the Trypan Blue (Gibco™) exclusion method on a Kova Slide (KOVA™). The isolated PBMCs were then used for subsequent experiments. For stimulation, freshly isolated PBMCs were seeded at 1 × 10^6^ cells/ml in 24-well tissue culture plates, with 1 ml of complete medium supplemented with 20 U/mL of IL-2 and 10 ng/mL of IL-7. The PBMCs were independently stimulated in triplicates with five peptides at a concentration of 20 µg/ml (P1: FAFLTTAIL, P2: TTAILVGMKW, P3: ALGAGCGVK, P4: GEDFLERR and P5: LLTETGAGRF). Positive controls included PMA (10 ng/ml) combined with Ionomycin (500 ng/ml) (data not shown) and HER2 (20 µg/ml), while sterile-filtered water was added to the culture medium as a negative control. Cells were incubated at 37°C with 5% CO_2_ for 6 days. On day 5, cells were re-stimulated with the peptides, and on day 6, all supernatants were collected and stored at -80°C for subsequent Enzyme-linked Immunosorbent Assays (ELISA).

### Interferon-gamma quantification by ELISA

Nunc MaxiSorp™ 96-well plates (Invitrogen™) were prepared by coating each well with 50µL of the capture antibodies. The plates were left to incubate overnight at room temperature. Following the incubation, the plates were washed twice with a wash buffer (PBS/Tween) and once with PBS. Subsequently, each well was blocked by the addition of 200µl of 1% PBS/BSA solution, followed by an incubation period of 1 h at room temperature. After the blocking step, the plates were washed, and 50µL of either 1/10 diluted samples or undiluted standards were added to the appropriate wells. The plates were then incubated for 2 h at room temperature. Post-incubation, the plates were washed, and 50µl of the detection antibody was added to each well. The plates were then incubated for an additional 2 hours at room temperature. After washing the plates, 50µl of Streptavidin-HRP was added to each well. The plates were then incubated in the dark for 20 minutes at room temperature. Following the incubation, the plates were washed, and 50µl of TmB substrate solution was added to each well. The plates were again incubated in the dark for 20 minutes at room temperature. The reaction was then stopped by adding 25µl of stop solution (2N H_2_SO_4_) to each well. The optical density of each well was assessed using a microplate reader Biotek Elx080 set to 450nm.

## Data Availability

The proteomics data presented in the study are deposited in the Proteome Xchange (PX) Consortium (37) via ProteomicsIdentifications (PRIDE) repository, accession number PXD057573. The sequencing data presented in the study are deposited in the National Center for Biotechnology Information (NCBI) repository, accession number PRJNA1235990: http://www.ncbi.nlm.nih.gov/bioproject/1235990.
