# Supplementary material for: Translation of bi-directional transcripts enhances MHC-I peptide diversity
Source: Front Immunol. 2025 Mar 17;16:1554561. doi: 10.3389/fimmu.2025.1554561 (PMC11956742; doi:10.3389/fimmu.2025.1554561)
Supplement: Supplementary file 1 [file DataSheet1.pdf]

SUPP. FIGURE 1.

A. SPECTRUM MATCH OF PEPTIDE AFLTTAILVGMK

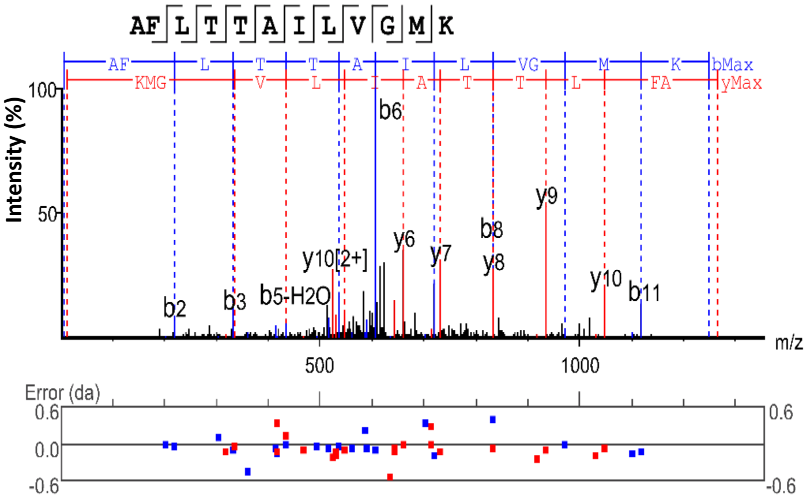

#	b	b-H2O	b-NH3	b (2+)	Seq	y	y-H2O	y-NH3	y (2+)	#
1	72.045	54.034	55.018	36.522	A					12
2	219.152	201.093	202.086	110.057	F	1193.696	1175.686	1176.669	597.348	11
3	332.299	314.187	315.170	166.599	L	1046.703	1028.804	1029.601	524.036	10
4	433.252	415.296	416.376	217.123	T	933.658	915.533	916.760	467.388	9
5	534.324	516.343	517.266	267.646	T	832.560	814.486	815.469	416.376	8
6	605.424	587.065	588.368	303.039	A	731.572	713.128	714.433	366.224	7
7	718.593	700.403	701.019	360.151	I	660.421	642.537	643.460	330.706	6
8	831.086	813.487	814.471	416.376	L	547.429	529.502	530.427	274.164	5
9	930.566	912.556	913.539	465.783	V	434.076	416.376	417.216	217.622	4
10	987.588	969.579	970.561	494.340	G	335.211	317.301	318.148	168.087	3
11	1118.764	1100.618	1101.774	559.882	M	278.153	260.143	261.126	139.577	2
12					K	147.113	129.102	130.086	74.056	1

B. SPECTRUM MATCH OF PEPTIDE FALGAGCGVK

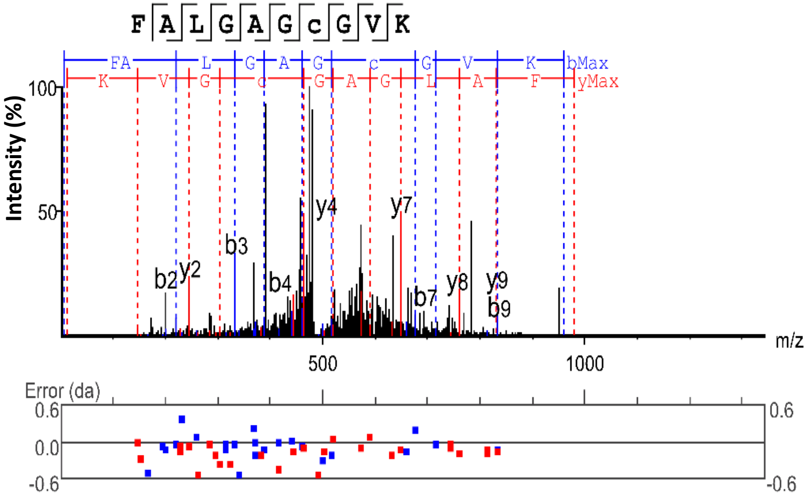

#	b	b-H2O	b-NH3	b (2+)	Seq	y	y-H2O	y-NH3	y (2+)	#
1	148.076	130.066	131.049	74.538	F					10
2	219.167	201.236	202.086	110.057	A	832.587	814.602	815.549	417.179	9
3	332.241	314.233	315.293	167.110	L	761.592	743.437	744.486	381.417	8
4	389.365	371.442	372.169	195.175	G	648.445	630.303	631.496	325.018	7
5	460.316	442.201	443.229	230.228	A	591.198	573.389	574.265	296.368	6
6	517.495	499.565	500.250	259.037	G	520.187	502.244	503.401	261.182	5
7	677.076	659.453	660.281	339.682	C(+57.02)	463.345	445.387	446.206	232.117	4
8	734.330	716.319	717.339	367.423	G	303.569	285.244	286.176	152.384	3
9	833.540	815.549	816.371	417.179	V	246.256	228.326	229.219	123.591	2
10					K	147.117	129.102	130.086	74.056	1

SUPP. FIGURE 1. (cont.)

C. SPECTRUM MATCH OF PEPTIDE ALGPGGGAGAR

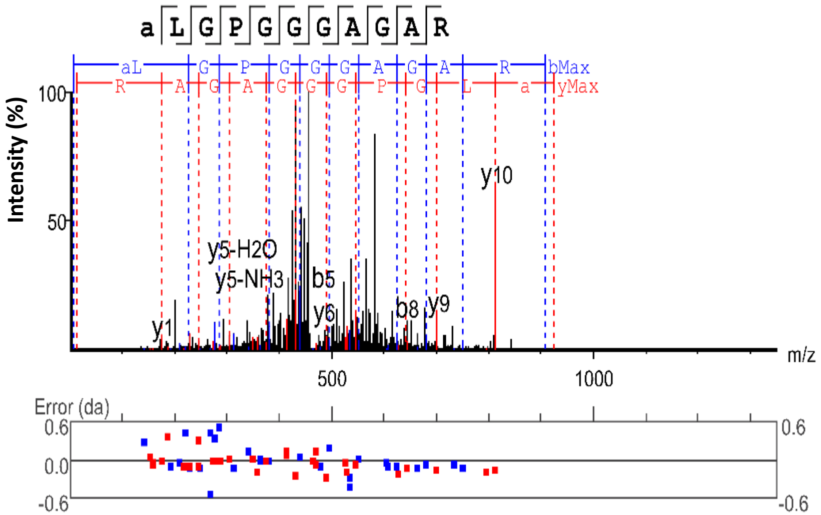

#	b	b-H2O	b-NH3	b (2+)	Seq	y	y-H2O	y-NH3	y (2+)	#
1	114.056	96.045	97.029	57.528	A(+42.01)					11
2	227.277	209.185	210.113	114.070	L	812.597	794.607	795.410	406.719	10
3	283.611	266.687	266.687	142.286	G	699.518	681.343	682.326	350.134	9
4	381.214	363.203	364.131	191.208	P	642.451	624.321	625.538	321.666	8
5	438.150	420.225	421.208	219.163	G	545.356	527.313	528.445	273.154	7
6	495.033	477.246	478.336	248.253	G	488.544	470.329	471.079	244.284	6
7	552.236	534.555	535.675	276.259	G	431.488	413.134	414.035	216.222	5
8	623.429	605.348	606.397	312.288	A	374.239	356.388	357.368	187.220	4
9	680.410	662.457	663.310	340.513	G	303.134	285.193	286.179	152.013	3
10	751.502	733.433	734.347	376.187	A	246.259	228.145	229.225	123.578	2
11					R	175.148	157.161	158.174	88.059	1

D. SPECTRUM MATCH OF PEPTIDE KTAAAAAAAAAAAAANR

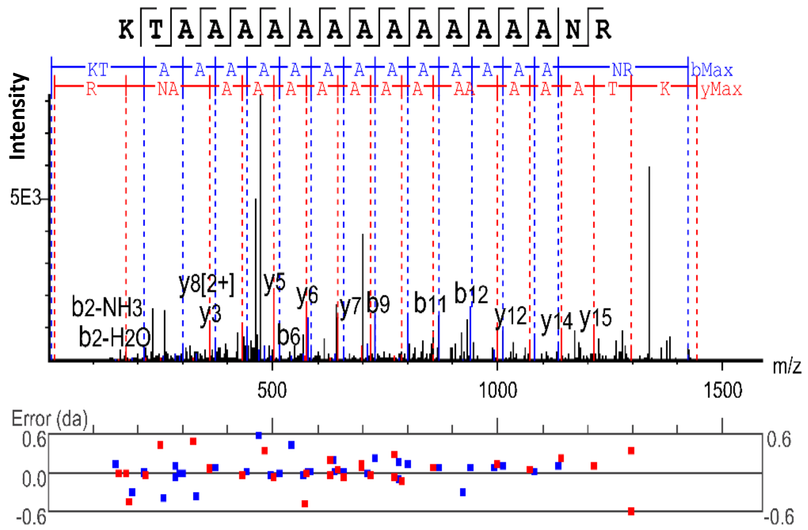

#	b	b-H2O	b-NH3	b (2+)	Seq	y	y-H2O	y-NH3	y (2+)	#
1	129.103	111.092	112.076	65.051	K					17
2	230.150	212.104	213.142	115.575	T	1313.692	1295.303	1297.253	657.405	16
3	301.180	283.050	284.247	150.930	A	1212.519	1194.634	1195.617	606.822	15
4	372.108	354.214	355.198	186.919	A	1141.369	1123.597	1124.580	571.799	14
5	443.223	425.251	426.235	222.131	A	1070.489	1052.560	1053.543	535.785	13
6	514.275	496.333	497.272	258.038	A	999.367	981.522	982.506	500.266	12
7	585.293	567.363	568.309	293.166	A	928.496	910.485	911.469	464.748	11
8	656.341	638.309	639.346	329.051	A	857.342	839.448	840.432	429.229	10
9	727.176	709.383	710.383	364.205	A	786.552	768.498	769.076	393.711	9
10	798.282	780.247	781.528	399.724	A	715.423	697.265	698.205	358.126	8
11	869.372	851.474	852.457	435.242	A	644.286	626.104	627.350	322.153	7
12	940.416	922.511	923.809	470.173	A	573.327	555.300	556.283	287.155	6
13	1011.425	993.447	994.532	506.279	A	502.347	484.263	485.246	251.183	5
14	1082.539	1064.585	1065.569	541.349	A	431.284	413.226	414.209	216.152	4
15	1153.633	1135.493	1136.606	577.283	A	360.099	342.188	343.172	181.053	3
16	1267.676	1249.665	1250.649	634.132	N	289.162	271.151	272.135	145.081	2
17					R	175.092	157.108	158.117	88.059	1

SUPP. FIGURE 1. (cont.)

E. **SPECTRUM MATCH OF PEPTIDE LGEDFLER**

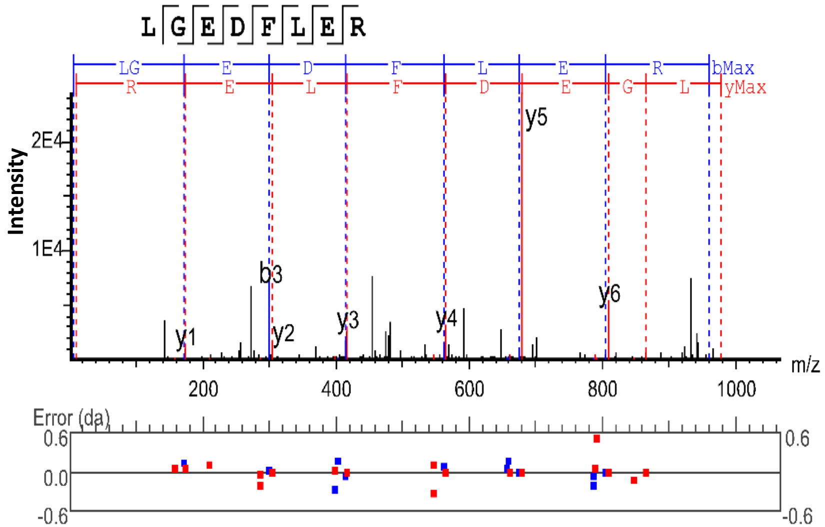

#	b	b-H2O	b-NH3	b (2+)	Seq	y	y-H2O	y-NH3	y (2+)	#
1	114.092	96.081	97.065	57.546	L					8
2	170.943	153.103	154.086	86.057	G	865.431	847.394	848.524	433.202	7
3	300.111	282.145	283.129	150.578	E	808.385	790.311	790.829	404.692	6
4	415.260	397.172	398.422	208.091	D	679.370	661.327	662.314	340.170	5
5	562.138	544.241	545.224	281.626	F	564.301	546.175	547.621	282.657	4
6	675.361	657.243	658.116	338.168	L	417.254	399.186	400.219	208.992	3
7	804.351	786.583	787.432	402.496	E	304.174	286.375	287.182	152.581	2
8					R	175.055	157.108	158.026	88.059	1

F. **SPECTRUM MATCH OF PEPTIDE TLLTETGAGR**

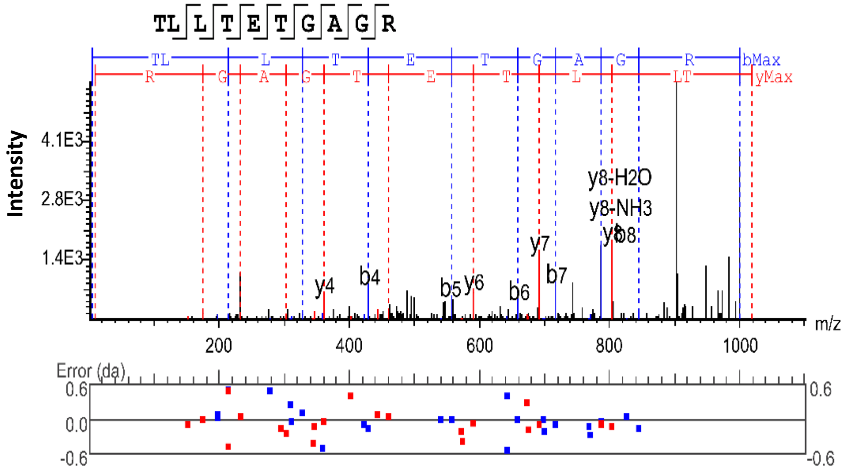

#	b	b-H2O	b-NH3	b (2+)	Seq	y	y-H2O	y-NH3	y (2+)	#
1	102.056	84.045	85.029	51.528	T					10
2	214.611	197.034	198.082	108.070	L	917.505	899.494	900.478	459.253	9
3	328.106	309.940	311.237	164.612	L	804.561	786.525	787.459	402.286	8
4	429.435	411.261	412.244	214.611	T	691.454	673.019	674.514	346.311	7
5	558.339	540.303	541.309	279.153	E	590.356	572.512	573.652	295.816	6
6	659.376	641.902	641.902	330.181	T	461.174	443.236	444.129	231.123	5
7	716.481	698.399	699.575	359.218	G	360.229	342.188	343.598	180.599	4
8	787.459	769.552	770.665	394.210	A	303.430	285.167	286.151	152.202	3
9	844.612	826.431	827.336	422.837	G	232.072	214.611	214.611	116.570	2
10					R	175.111	157.108	158.092	88.059	1

SUPPLEMENTARY FIGURE 1. Na-PTP peptides identified from Hek293 cells. Na-PTP peptides identified from Hek293 cells. (A). Spectrum match of peptide AFLTTAILVGMK from sample 49-4B. Scan n. 33098; PEAKS score: 33.61; mass 1263.7261 Da; Precursor error -1.2 ppm; Retention time 46.04 min. **(B).** Spectrum match of peptide FALGAGCGVK from sample 49-7A. Scan n. 17075; PEAKS score: 34.72; mass 978.4957 Da; Precursor error -0.2 ppm; Retention time 27.96 min. **(C).** Spectrum match of peptide ALGPGGGAGAR from sample 49-7A. Scan n. 15840; PEAKS score: 32.97; mass 924.4777 Da; Precursor error 0.7 ppm; Retention time 26.83 min. **(D).** Spectrum match of peptide KTAAAAAAAAAAAAANR from sample 10-2B. Scan n. 24968; PEAKS score: 50.09; mass 1440.78 Da; Precursor error -6 ppm; Retention time 36.63 min. **(E).** Spectrum match of peptide LGEDFLER from sample 10-1B. Scan n. 20145; PEAKS score: 34.28; mass 977.4818 Da; Precursor error 2.5 ppm; Retention time 33.27 min. **(F).** Spectrum match of peptide TLLTETGAGR from sample 73-21 (Hek293). Scan n. 7058; PEAKS score: 36.25; mass 1017.5455 Da; Precursor error 1 ppm; Retention time 17.32 min.The Orbitrap Fusion mass spectrometer was used for the measurement. Relates to Figure 2.

SUPP. FIGURE 2.

A. **SPECTRUM MATCH OF PEPTIDE TLLTETGAGR (H1299 cells)**

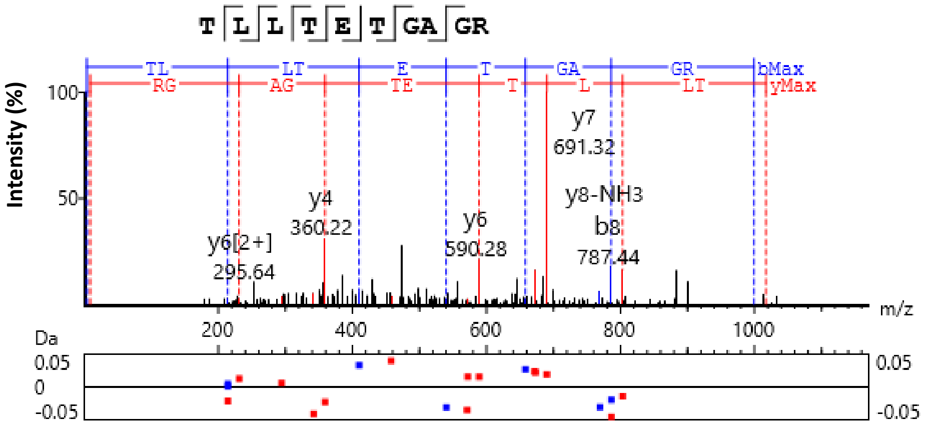

#	b	b-H2O	b-NH3	b(2+)	Seq	y	y-H2O	y-NH3	y(2+)	#
1	102.06	84.04	85.03	51.53	T					10
2	215.14	197.13	198.11	108.07	L	917.51	899.49	900.48	459.21	9
3	328.22	310.21	311.20	164.61	L	804.44	786.41	787.44	402.71	8
4	429.27	411.23	412.24	215.14	T	691.32	673.30	674.29	346.17	7
5	558.31	540.30	541.32	279.66	E	590.28	572.32	573.25	295.64	6
6	659.34	641.35	642.33	330.18	T	461.25	443.24	444.22	231.12	5
7	716.38	698.37	699.36	358.69	G	360.22	342.19	343.21	180.60	4
8	787.44	769.41	770.42	394.21	A	303.18	285.17	286.15	152.09	3
9	844.44	826.43	827.41	422.72	G	232.13	214.13	215.14	116.57	2
10					R	175.12	157.11	158.09	88.06	1

SUPPLEMENTARY FIGURE 2. Na-PTP peptides identified from H1299 cells) (A). Spectrum match of peptide TLLTETGAGR from sample 02-3 (H1299). Precursor Id. 26826; PEAKS score: 26.85; mass 1017.5455 Da; Precursor error -4.4 ppm; Retention time 22.26 min. The TimsTOF SCP mass spectrometer was used for the measurement. Relates to Figure 2.

SUPP. FIGURE 3.

A.

TTT GCC TTT TTG ACT ACT GCC ATC CTA GTG GGT ATG AAG TGG

F A F L T T A I L V G M K W

tttgcctttttgactactgccatcctagtgggtatgaagtggtgtatcattgtgcttttgatttacattaccataatggctaatgataagagcatctttttatatgtctattggcc
tttttttttttttttttttttttttgagacggagtctcactctgttgcccaggctggagtgcagtggcgcgatctctgctcactgcaagctccacctcccgggttcatgccattctcc
tgccttagccttccaagtagctgggactacaggcgcccgccaccatgcccagctaatttttgtatttttagtagagacagggtttcaccgtgttggccaggatggtctcgat
ctcctgacctcatgatctgcctgcctcggcctcccaaagtgctgggattacaggcgtga

FAFLTTAILVGMKWCIIVLLIYITIMANDKSIFLYVYWPFFFFFFFLRRSLTLLPRLECSGAISAHCKLHLPGSCHSPALAFQVAGTTGARHHAQLIFCIFSRDRVSPCWPG
WSRSPDLMICLPRPPKVLGLQA **(133aa)**

B.

CGC TTT GCA CTG GGG GCT GGA TGT GGT GTG AAG GCT

R F A L G A G C G V K A

cgctttgcactgggggctggatgtggtgtgaaggctgcgcccacgaggcccggtcccgggctgcagcgctgtggagctcggctccgccctggggagagctcccacccatc
gctcgctctccaccccaaattctctctcctcctgggcctggggcggtggccctga

RFALGAGCGVKAAPTRPGPGLQRCGARLRPGESSHPSLALHPKFSLLLGLGRWP **(53aa)**

C.

CAA AAA ACT GCT GCT GCT GCT GCT GCT GCT GCT GCT GCT GCT GCT GCT AAC AGA ATG

Q K T A A A A A A A A A A A A A N R M

caaaaaactgctgctgctgctgctgctgctgctgctgctgctgctgctaacagaatgactttacagcatgttcttttcatgcccattgtttgttttgccaagccatctaagag
catgagcttaattacttcatactaa

QKTAAAAAAAAAAAAANRMTLQHVLFMPIVCFAKPSKSMSLITSY **(45aa)**

SUPP. FIGURE 3. (cont.)

D.

GCG CTC GGG CCG GGC GGG GGC GCC GGG GCC CGG GGG

A L G P G G G A G A R G

gcgctcgggccgggcgggggcgccggggcccgggggcgggaagggggaggccaggacgaggaggggcgcgcggcggcggggccgggccggggtctgcgggcgggggaggggcggccc
ggcgggcgcggctcgggggagggtccgttccaggggccgcgcccgcgggctggggttctttgtcagcggcgctgcggcgagatccggacaaaggaggcggcggggggcggaggccgctttgtgg
ggatcgcggggcgctcgcccccgcccgtgcagcccccgcccgtcctcagctacgctcccgccttcccccggcatttcggggttcttcaaaattctgggagctccggggagggctccggggataggggt
ccaggtgacgtccggcgcccgtctcgcgggtagggaaactgaggccgggccgcagcgttcggcgcccggttccgtgggcagcggctgcgcggcgtgggcatgcctagctcccctggtccccgtccc
acaccccgcctccgtcgggccgattcgagctacctccccagctgccgtcccctctccagctgtcgcccccctgggccgagccccctcctccctggagcctcgctttgctcctccgtggagtgggggagac
gccggcgccgccgggcagggtggaacgggagggtgcgcggagcgcgcctggcaccgcgcctgccacgccgcccgcgctcagcactcgggagccgccggggcgctgtcacccctaa

ALGPGGGAGARGREGGGQDEEGRARRRGRAGVCGRGRGGPAGAARGRVRSRGRARGLGFFVSGAAARSGQRRRRGAEAALWGSRGARPRPCSPRPSS
ATLPPSPGISGFFKILGAPGRAPGIGVQVTSGARLAGRETEAGPQRSAPGSVGSGCAAWACLAPLVPVPHPASVGPIRATSPAAVPSPAVAPWAEPPPPWSL
ALLLRGVGETPAPPGRVEREGARSAPGTAPATPPALSTREPPGRCHP **(246aa)**

E.

AGA CTA GGA GAA GAT TTT CTG GAG CGG AGA

R L G E D F L E R R

agactaggagaagattttctggagcggagagacttgagcacactttggaaggtggtgttgaagttagccgggccatgggagctgggcaggtttcctgggcctcctgtttgccacaccacatctctgtcttcttc
ctccagctctgcaccagcgcaggcgaccgtagacactgtacctgtgtcccatgccttccctgagcctgtgggcagcactggctgtcaggttggagggagggagaggatgcgtatgaggtgtttcctagctctg
gggtgctgcactttccgttttgcttctccctttcattacaagccctgcccacacctttgtaaacacttcctttctcagctcacctcagttccccagctggcgtcccaggcagaggcagttgcatgtgcaaaggccc
ggagccaggacttgataaatatggctcaagatgctgcagcctag

RLGEDFLERRDLSTLWKVVLKLAGPWELGRFPGPPVCHTTSLSSSSSAPAQATVDTVPVSHAFPEPVGSTGCQVGGRERMRMRCFLALGCCTFRFASPFHYKPCP
HLCKHFLSQLTSVPQLASQAEAVACAKARSQDLINMAQDAAA **(147aa)**

F.

AAA ACT TTA TTA ACA GAA ACA GGT GCT GGG AGA TTT

K T L L T E T G A G R F

aaaactttattaacagaaacaggtgctgggagatttagtccatgggctctagttgcccaactgtga

KTLLTETGAGRFSPWALVAQL **(20aa)**

<u>SUPPLEMENTARY FIGURE 3</u>. Na-PTP peptides (green) from Hek293 cells identified by mass spectrometry analysis and verified by SFDB. The peptides and corresponding genomic sequences are shown. Below is shown the sequence until stop codon to calculate minimal Na-PTP length. Red indicates flanking amino acids used for predicting MHC-I binding peptides. **(A)** Peptide generated from anti-sense strand of gene CWF19L1. **(B)** Peptide generated from anti-sense strand of gene IQSEC2. **(C)** Peptide generated from intron 24 of gene LRRC7. Relates to figure 2 and Table 1. **(D)** Peptide generated from intron 1 of gene SNN. **(E)** Peptide derived from intron two of gene SPECC1. **(F)** Peptide derived from anti-sense strand of gene ZNF615. Note that peptide TLLTETGAGR was also detected from anti-sense strand of gene ZNF615 in H1299 cells. Relates to Figure 2 and Table 1.

SUPP. FIGURE 4.

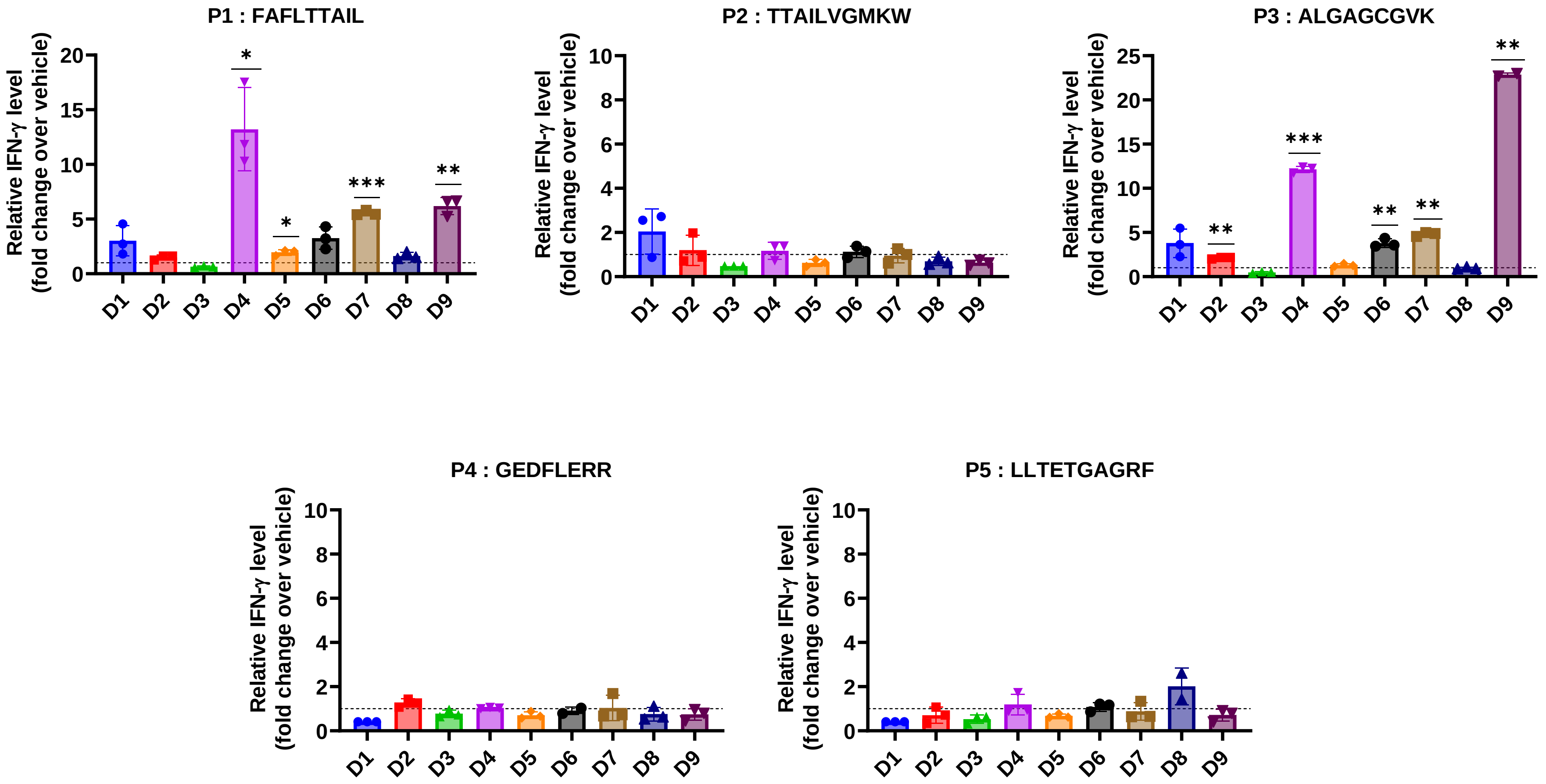

SUPPLEMENTARY FIGURE 4. IFN-γ production by CD8+ T cells from PBMCs of individual healthy donors in response to Na-PTP peptides. Relative IFN-γ levels (fold change over vehicle) for each donor (D1 to D9) upon stimulation with peptides P1 (FAFLTTAIL), P2 (TTAILVGMKW), P3 (ALGAGCGVK), P4 (GEDFLERR), and P5 (LLTETGAGRF). Each panel represents the response to a specific peptide across donors, highlighting donor-specific IFN-γ production. Statistical significance was determined using a one-sample t-test comparing each donor's response to a theoretical mean of 1. Differences were considered statistically significant when $P < 0.05$. Relates to Figure 4.

SUPP. FIGURE 5.

IMMUNE RESPONSE ASSAYS

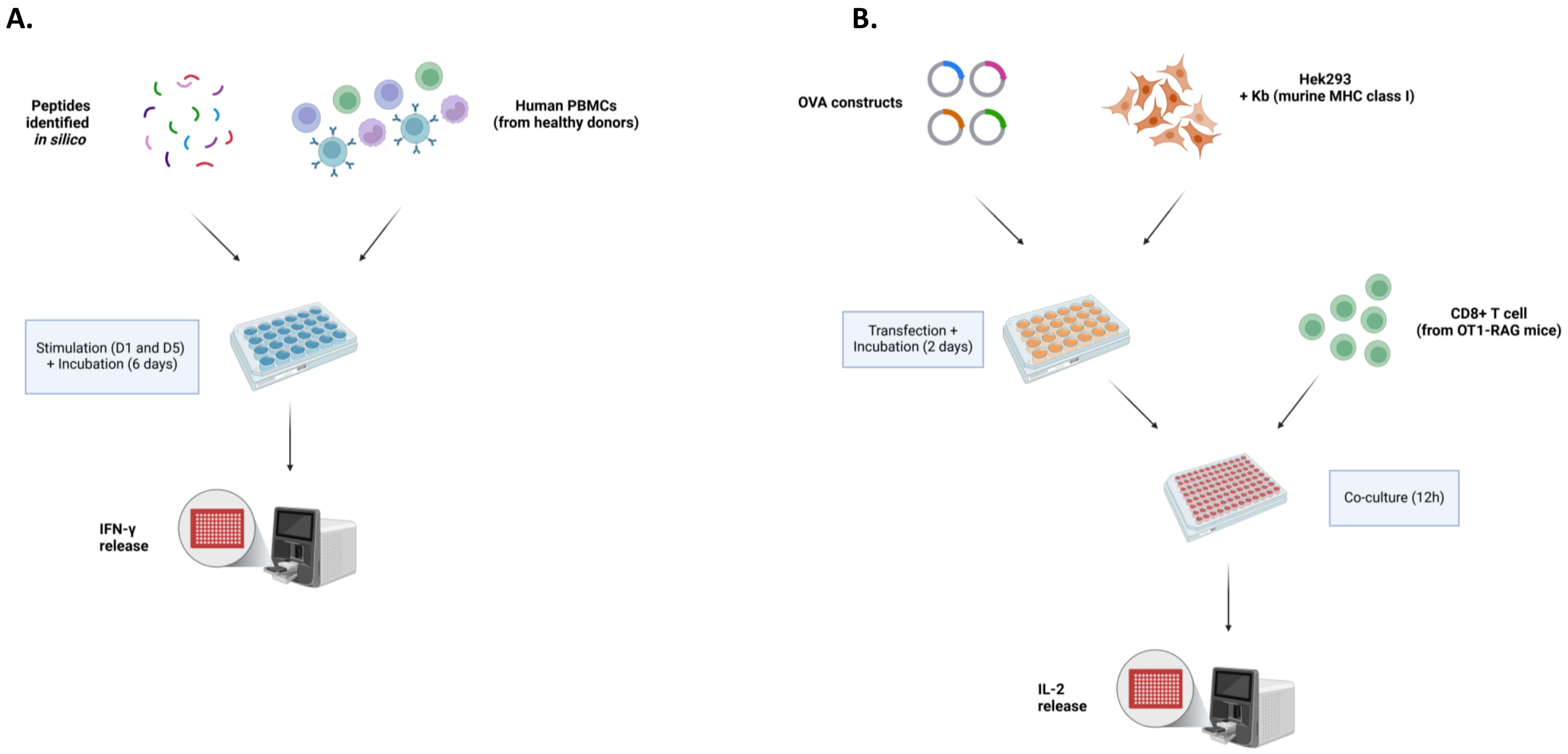

<u>SUPPLEMENTARY FIGURE 5.</u> (A). Immune response assay to peptide stimulation. PBMCs were isolated from buffy coats using a Ficoll gradient, cultured in complete medium with IL-2 and IL-7, and stimulated with specific peptides for 6 days (stimulation at days 1 and 5) to assess immune responses. IFN-γ levels in collected supernatants were quantified via ELISA. **(B).** Antigen presentation assay. Transiently co- transfected Hek293 cells with Kb and OVA constructs are co-cultured for 12h with OT-1 CD8+ T cell isolated from OT1-RAG mice. IL-2 concentration in the medium from co-cultured cells is analyzed by ELISA and is used as an indirect measure of antigenic peptide substrate synthesis. This figure was created in BioRender. Habault, J. (2024) https://BioRender.com/w63q319. Relates to Figures 4 and 5.

SUPP. FIGURE 6.

A.

HPLC QC OF PEPTIDE GEDFLERR

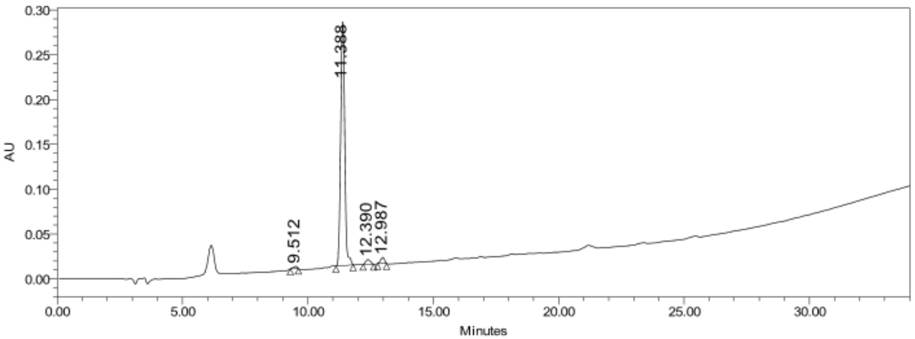

D.

HPLC QC OF PEPTIDE SPVSASTL

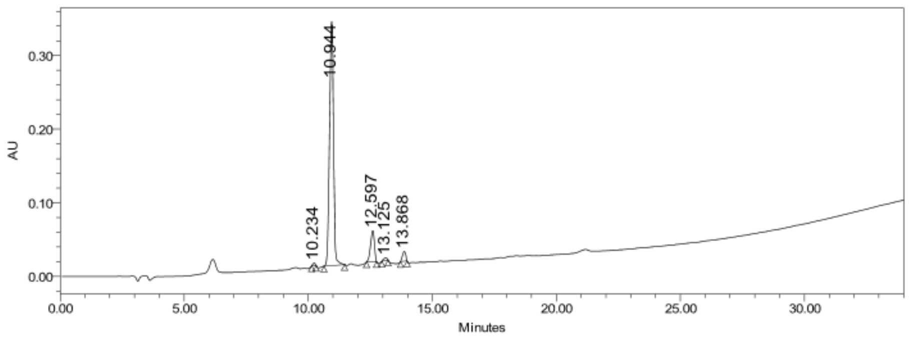

B.

HPLC QC OF PEPTIDE LLTETGAGRF

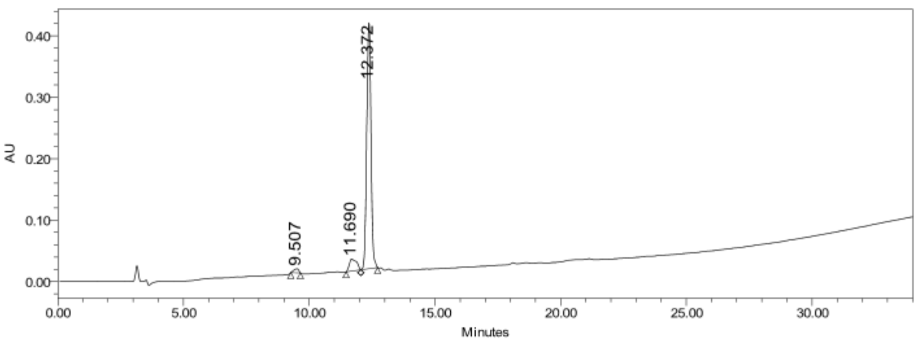

E.

HPLC QC OF PEPTIDE DETGAKVEL

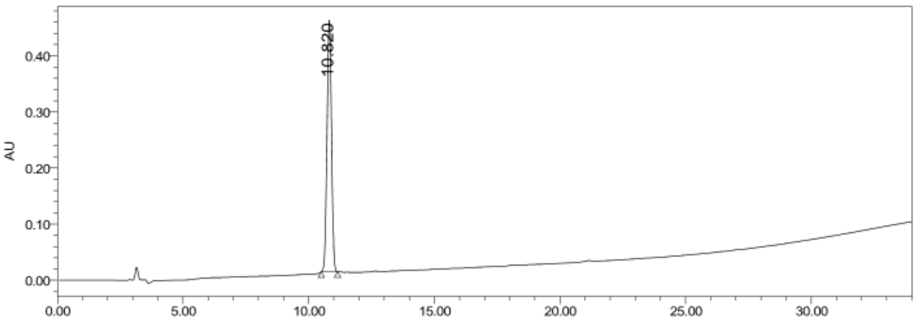

C.

HPLC QC OF PEPTIDE ALGAGCGVK

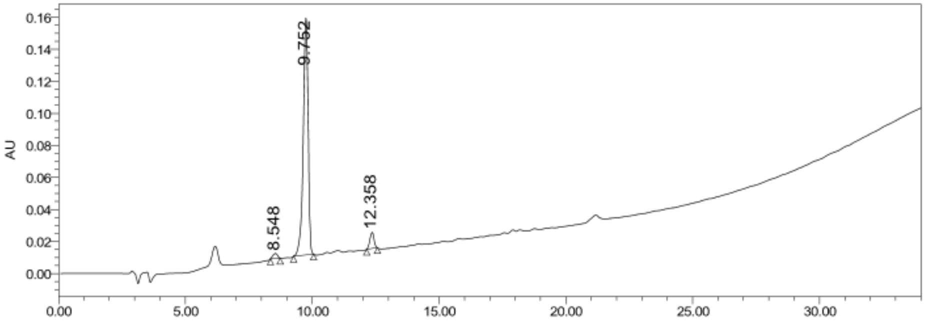

F.

HPLC QC OF PEPTIDE FAFLTTAIL

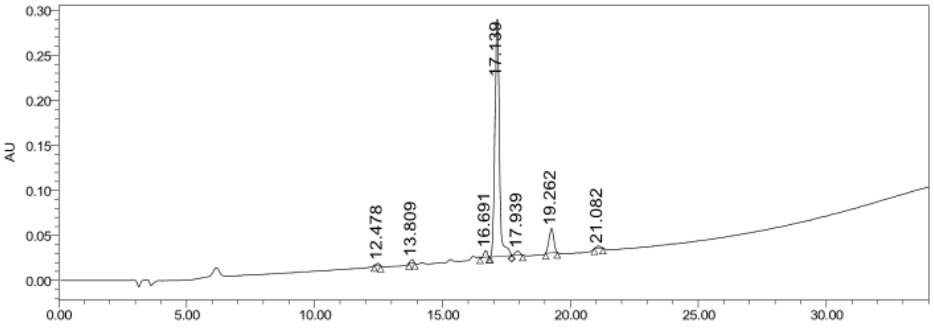

SUPP. FIGURE 6. (cont.)

G.

HPLC QC OF PEPTIDE TTAILVGMKW

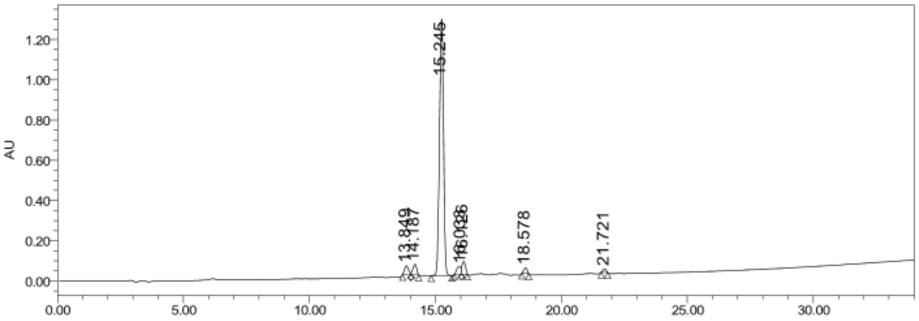

SUPPLEMENTARY FIGURE 6. Verification of the purity of synthetic peptides by high-performance liquid chromatography (HPLC). Representative chromatograms of the synthetic peptides show a single major peak, confirming their purity (A–G). Relates to Figure 4.

SUPP. TABLE 1.

Primer	Sequence (5' > 3')
**CWF19L1**	
CWF19L1_Fw1	GATGCTCTTATCATTAGCCATTATG
CWF19L1_Fw2	GCACAATGATACACCACTTC
CWF19L1_Rev	GATTCCAATTCCTCCACATCC
**IQSEC2**	
IQSEC2_Fw1	GCTCCACAGCGCTGCAGC
IQSEC2_Fw2	GCCTCGTGGGCGCAGCC
IQSEC2_Rev	CAGTGCAGGAGCTGGAACTG
**OVA**	
OVA-Fw	GATGAAGACACACAAGCAAT
OVA-Rev	AAGCCATTGATGCCACTCTA
**β-Actin**	
Actin-Fw	TCACCCACACTGTGCCCATCTACGA
Actin-Rev	TGAGGTAGTCAGTCAGGTCCCG

SUPPLEMENTARY TABLE 1. Specific primers used for RT-qPCR to evaluate the sense or anti-sense transcription of the CWF19L1 and IQSEC2 (peptides) or OVA (model) genes. Actin amplification was used to normalize data. Relates to Figures 3 and 5.
